# Supplementary material for: Interferons and viruses induce a novel primate-specific isoform dACE2 and not the SARS-CoV-2 receptor ACE2
Source: bioRxiv. 2020 Jul 20:2020.07.19.210955. Preprint. [Version 1] doi: 10.1101/2020.07.19.210955 (PMC7386494; doi:10.1101/2020.07.19.210955)
Supplement: Supplement 2 [file media-2.pdf]

SUPPLEMENTARY MATERIALS

Onabajo, Banday et al,

Supplementary Tables

Table S1. Expression of IFNs and select ISGs in T47D cells at baseline and after SeV infection, RNA-seq FPKM, hg38

Gene	IFN type	Untreated 1	Untreated 2	SeV 12hrs.1	SeV 12hrs.2
IFNA1	Type I	0.00	0.00	0.52	0.27
IFNA2	Type I	0.00	0.00	0.13	0.04
IFNA4	Type I	0.00	0.00	0.00	0.00
IFNA5	Type I	0.00	0.00	0.00	0.00
IFNA6	Type I	0.00	0.00	0.00	0.00
IFNA7	Type I	0.00	0.00	0.30	0.25
IFNA8	Type I	0.00	0.00	0.00	0.05
IFNA10	Type I	0.00	0.00	0.24	0.39
IFNA13	Type I	0.00	0.00	0.33	0.09
IFNA14	Type I	0.00	0.00	0.00	0.08
IFNA16	Type I	0.00	0.00	0.00	0.11
IFNA17	Type I	0.00	0.00	0.00	0.00
IFNA21	Type I	0.00	0.00	0.00	0.05
**IFNB1**	**Type I**	**0.00**	**0.00**	**337.30**	**381.62**
IFNE	Type I	0.00	0.00	0.04	0.03
IFNK	Type I	0.00	0.00	0.00	0.00
IFNG	Type II	0.00	0.00	0.00	0.00
**IFNL1**	**Type III**	**0.00**	**0.00**	**252.53**	**292.93**
**IFNL2**	**Type III**	**0.00**	**0.00**	**110.41**	**123.48**
**IFNL3**	**Type III**	**0.00**	**0.00**	**122.21**	**138.66**
**IFNL4**	**Type III**	**0.00**	**0.00**	**122.77**	**132.11**
ISG15	ISG	4.29	3.25	2698.66	3206.30
IFIT1	ISG	0.94	0.81	879.53	1033.89
MX1	ISG	5.20	4.66	849.95	881.69

FPKM - fragments per kilobase of exon per million reads mapped

Table S2. Expression of *ACE2* and *dACE2* in various cell lines and conditions (separate Excel file)

Table S3. Cell lines used

Cells	Cell type	Source	Media
Primary tonsil epithelial cells	Normal tissue from donors	ScienCell	Tonsil Epithelial Cell Medium
T47D (MDA-MB-23)	Breast cancer	ATCC	DMEM
T24	Bladder cancer	ATCC	McCoy's 5A
HT-1376	Bladder cancer	ATCC	DMEM
HTB-9	Bladder cancer	ATCC	RPMI-1640
RT-4	Bladder cancer	ATCC	McCoy's 5A
HBLAK	Immortalized uroepithelial	CELLnTEC	CnT-Prime
PC3	Prostate cancer	ATCC	F-12
22RV1	Prostate cancer	ATCC	RPMI
DU145	Prostate cancer	ATCC	EMEM
HepG2	Liver cancer	ATCC	DMEM
Caco-2	Colon cancer	ATCC	EMEM
T84	Colon cancer	ATCC	DMEM: F-12
A549	Lung cancer	ATCC	F-12
Calu3	Lung cancer	ATCC	DMEM
Capan-1	Pancreatic cancer	ATCC	IMDM
HeLa	Cervical cancer	ATCC	EMEM
TCCSUP/HTB5	Bladder Cancer	ATCC	EMEM
5637/HTB9	Bladder Cancer	ATCC	RPMI
J82	Bladder Cancer	ATCC	EMEM
SW780	Bladder Cancer	ATCC	Leibovitz's L-15 Medium
UMUC3	Bladder Cancer	ATCC	EMEM
293T	Kidney	ATCC	DMEM
NHBE	Primary normal human bronchial epithelial cells from 5 donors Described in (Santer et al., 2020)	International Institute for the Advancement of Medicine	BEGM (bronchial epithelial growth medium) + bulletkit
Organoid cultures of colon and ileum	Described in (Stanifer et al., 2020)	University Hospital Heidelberg	Human organoid media

ATCC - American Type Culture Collection

Table S4. Primers and expression assays used

Primers	Sequence	Assay type, amplicon size
ACE2_F	GGGCGACTTCAGGATCCTTAT	ACE2 SYBR Green assay, 80 bp
ACE2_R	GGATATGCCCCATCTCATGATG	
dACE2_F	GGAAGCAGGCTGGGACAAA	dACE2 SYBR Green assay, 73 bp
dACE2_R	AGCTGTCAGGAAGTCGTCCATT	
ACE2_F	GGGCGACTTCAGGATCCTTAT	ACE2 TaqMan assay, 80 bp
ACE2_R	GGATATGCCCCATCTCATGATG	
ACE2_probe	ATGGACGACTTCCTGACAG	
dACE2_F	GGAAGCAGGCTGGGACAAA	dACE2 TaqMan assay, 73 bp
dACE2_R	AGCTGTCAGGAAGTCGTCCATT	
dACE_probe	AGGGAGGATCCTTATGTG	
dACE2_F	AGTGCTTCATTGAGGAGAGCTCT	dACE2, 5'-3'UTR, 1535 bp 98°C-30s, 98°C-10s, 60°C-30s, 72°C-40s, 35 cycles, 72°C-2 min; Q5 High-Fidelity 2X PCR Master Mix (NEB)
dACE2_R	TCTATACCATGAAATTAACATTTACATACAAC	
HPRT1_F	TGACACTGGCAAAACAATGCA	SYBR Green assay, 94 bp
HPRT1_R	GGTCCTTTTCACCAGCAAGCT	
MX1_F	ACCTGATGGCCTATCACCAG	SYBR Green assay, 154 bp
MX1_R	TTCAGGAGCCAGCTGTAGGT	
IFIT1_F	AAAAGCCCACATTTGAGGTG	SYBR Green assay
IFIT1_R	GAAATTCCTGAAACCGACCA	SYBR Green assay
GAPDH	Hs04420632_g1 (Thermo Fisher)	TaqMan assay
ACTB	4352667 (Thermo Fisher)	
ISG15	Hs01921425_s1 (Thermo Fisher)	TaqMan assay

Table S5. Reagents used

Antibodies						
**Target gene**	**Cat. No.**	**Source**	**Target species**	**Host**	**Tag**	**Dilution**
ACE2	ab15348	Abcam	Human	Rabbit		1:250
Myc-DDK		Thermo Fisher	Tag	Rabbit		1:1000
GAPDH	Ab9485	Abcam	Human	Rabbit		1:1000
GFP	MA515256	Thermo Fisher	Tag	Mouse		1:1000
DYKDDDDK Epitope Tag	NB600-347	Novus Biologicals	Tag	Goat		1:1000
IgG	#7074	Cell Signaling	Rabbit	Goat	HRP	1:5000
IgG	sc2314	Santa Cruz	Mouse	Donkey	HRP	1:5000
IgG	sc2304	Santa Cruz	Goat	Donkey	HRP	1:5000
Streptavidin	SA10044	Thermo Fisher	Tag		PE	1:200
IgG	A32734	Thermo Fisher	Rabbit	Goat	AF680	1:200

Interferons				
**IFN**	**Source**	**Concentration**	**Time**	**Experiment**
IFNa2b	Merck, Intron A	100 IU/ml	24 hrs	NHBE
IFN-λ3	R&D Systems, Cat# 5259-IL/CF	100 ng/ml	24 hrs	NHBE
IFN-β1	Biomol, Cat#86421	2000IU/mL	24 hrs	Organoids Cell lines
IFN-λ1	Peprotech, Cat#300-02L	100 ng/ml	24 hrs	Organoids Cell lines
IFN-λ2	Peprotech, Cat#300-02K	100 ng/ml	24 hrs	Organoids Cell lines
IFN-λ3	Biomol, Cat#179-ML-025	100 ng/ml	24 hrs	Organoids Cell lines
IFN-β	R&D Systems, Cat# 8499-IF	0.5 ng/ml	48 hrs	Cell lines
IFN-γ	R&D Systems Cat# 285-IF	2 ng/ml	48 hrs	Cell lines

Table S6. RNA-seq datasets analyzed

Datasets	NCBI SRA	Alignment reference genome	Reference
Breast cancer cell line T47D, SeV-infected for 12 hours (n=2), not infected, n=2	PRJNA512015	hg19	Current work
Nasal epithelial cells from 30 asthmatic patients were infected with rhinovirus strains - RV-A16 (n=30), RV-C15 (n=30) or not infected (n=30)	PRJNA627860	hg38	NA
Human lung explants infected with influenza A/H3N2 virus from 5 donors, n=20	PRJNA557257	hg38	NA
Lung cells infected with the respiratory syncytial virus (RSV): human lung mucoepidermoid pulmonary carcinoma cell line H292, RSV-infected (n=1) and mock (n=1); lung cells from mice infected with RSV, n=3 and mock, n=3	PRJNA588982	hg38 and mm10	(McAllister et al., 2020)
Normal human tissues, n = 95	PRJEB4337	hg38	(Fagerberg et al., 2014)

Table S7. Nucleotide sequences and genome coordinates of three alternative first exons of *ACE2* and *dACE2* used for quantification of RNA-seq reads

Exon	Sequence	Coordinates, hg38	Length, bp	RefSeq ID
ACE2, Ex1a	GGCACTCATACATACACTCTGGCA<br>ATGAGGACACTGAGCTCGCTTCTG<br>AAATTTGACAAGATAACCACTAAA<br>ATCTCTTTGAATTCTATGTTGTTGT<br>GATCCCATGGCTACAGAGGATCAG<br>GAGTTGACATAGATACTCTTTGGAT<br>TTCATACCATGTGGAGGCTTTCTTA<br>CTTCCACGTGACCTTGACTGAGTTT<br>TGAATAG	chrX:15,601,956-15,602,158	203 bp	NM_021804.3
ACE2, Ex1b	CGCCCAACCCAAGTTCAAAGGCTG<br>ATAAGAGAGAAAATCTCATGAGGA<br>GGTTTTAGTCTAGGGAAAGTCATTC<br>AGTGGATGTGATCTTGGCTCACAG<br>GGGACGATGTCAAGCTCTTCCTGG<br>CTCCTTCTCAGCCTTGTTGCTGTAA<br>CTGCTGCTCAGTCCACCATTGAGG<br>AACAGGCCAAGACATTTTTGGACA<br>AGTTTAACCACGAAGCCGAAGACC<br>TGTTCTATCAAAGTTCACTTGCTTC<br>TTGGAATTATAACACCAATATTACT<br>GAAGAGAATGTCCAAAACATG	chrX:15,600,726-15,601,014	289 bp	NM_021804
dACE2, Ex1c	GTAATTCCCAGGTTGCAGGCTT<br>GTGAGAGCCTTAGGTTGGATTC<br>CCTAGCTTGAAAAGGAGATCGT<br>TTTACAAGTGCTTCATTGAGGA<br>GAGCTCTGAGGCAGAGGGGAA<br>TGAGGGAAGCAGGCTGGGACA<br>AAGGAGGGAG	chrX:15,580,281-15,580,420	140 bp	MT505392

Supplementary Figures

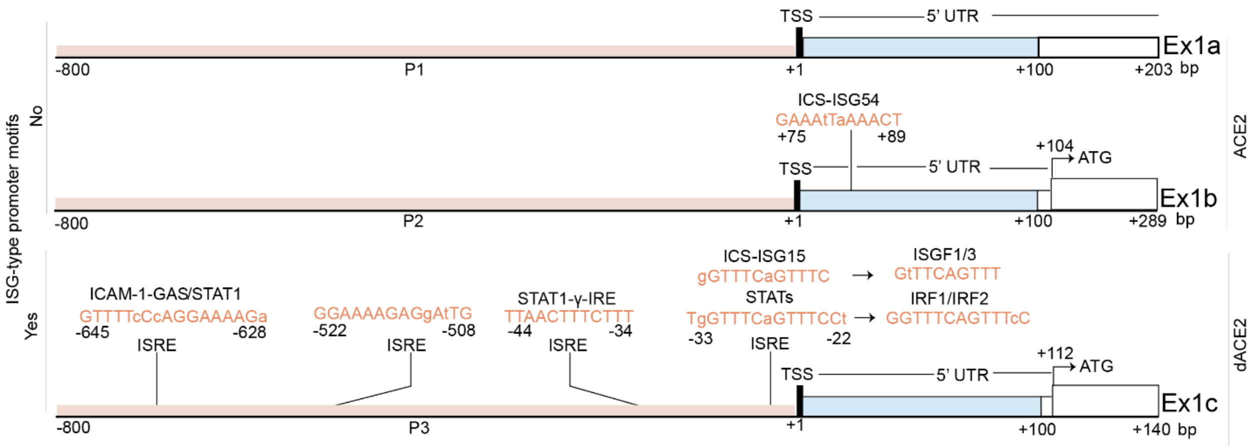

Figure S1. Analysis of promoter regulatory elements relevant for IFN signaling

Promoters of *ACE2* (P1 and P2) and *dACE2* (P3) were analyzed for binding motifs of transcription factors relevant for IFN signaling. Promoters were defined within the -800 bp/+100 bp window from the corresponding transcription start sites (TSS).

A

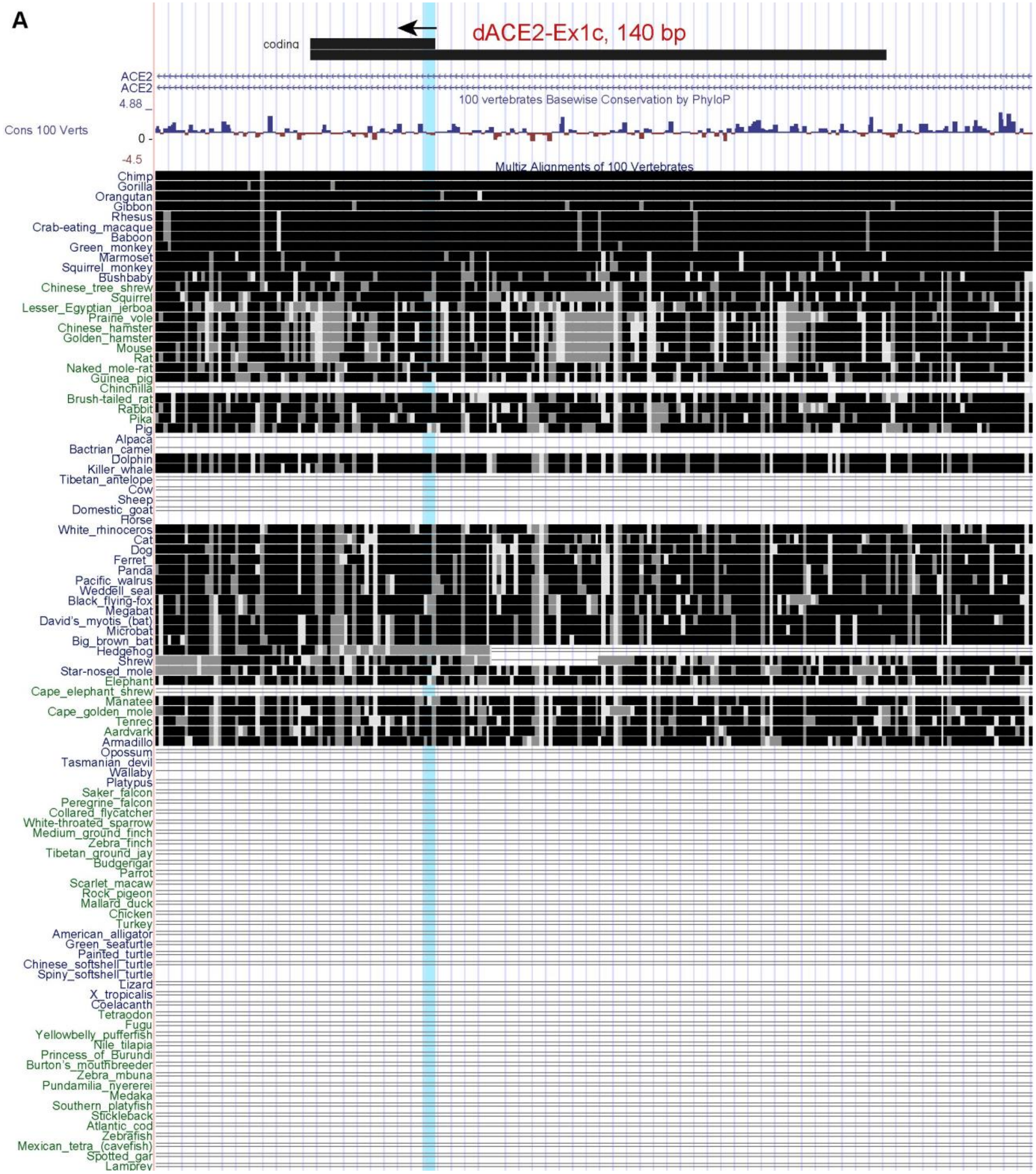
dACE2-Ex1c, 140 bp
coding
ACE2
ACE2
4.88
100 vertebrates Basewise Conservation by PhyloP
Cons 100 Verts
0
-4.5
Multiz Alignments of 100 Vertebrates
Chimp
Gorilla
Orangutan
Gibbon
Rhesus
Crab-eating_macaque
Baboon
Green_monkey
Marmoset
Squirrel_monkey
Bushbaby
Chinese_tree_shrew
Squirrel
Lesser_Egyptian_jerboa
Prairie_vole
Chinese_hamster
Golden_hamster
Mouse
Rat
Naked_mole-rat
Guinea_pig
Chinchilla
Brush-tailed_rat
Rabbit
Pika
Pig
Alpaca
Bactrian_camel
Dolphin
Killer_whale
Tibetan_antelope
Cow
Sheep
Domestic_goat
Horse
White_rhinoceros
Cat
Dog
Ferret
Panda
Pacific_walrus
Weddell_seal
Black_flying-fox
Megabat
David's_myotis_(bat)
Microbat
Big_brown_bat
Hedgehog
Shrew
Star-nosed_mole
Elephant
Cape_elephant_shrew
Manatee
Cape_golden_mole
Tenrec
Aardvark
Armadillo
Opossum
Tasmanian_devil
Wallaby
Platypus
Saker_falcon
Peregrine_falcon
Collared_flycatcher
White-throated_sparrow
Medium_ground_finch
Zebra_finch
Tibetan_ground_jay
Budgerigar
Parrot
Scarlet_macaw
Rock_pigeon
Mallard_duck
Chicken
Turkey
American_alligator
Green_seaturtle
Painted_turtle
Chinese_softshell_turtle
Spiny_softshell_turtle
Lizard
X_tropicalis
Coelacanth
Tetraodon
Fugu
Yellowbelly_pufferfish
Nile_tilapia
Princess_of_Burundi
Burton's_mouthbreeder
Zebra_mbuna
Pundamilia_nyererei
Medaka
Southern_platyfish
Stickleback
Atlantic_cod
Zebrafish
Mexican_tetra_(cavefish)
Spotted_gar
Lamprey

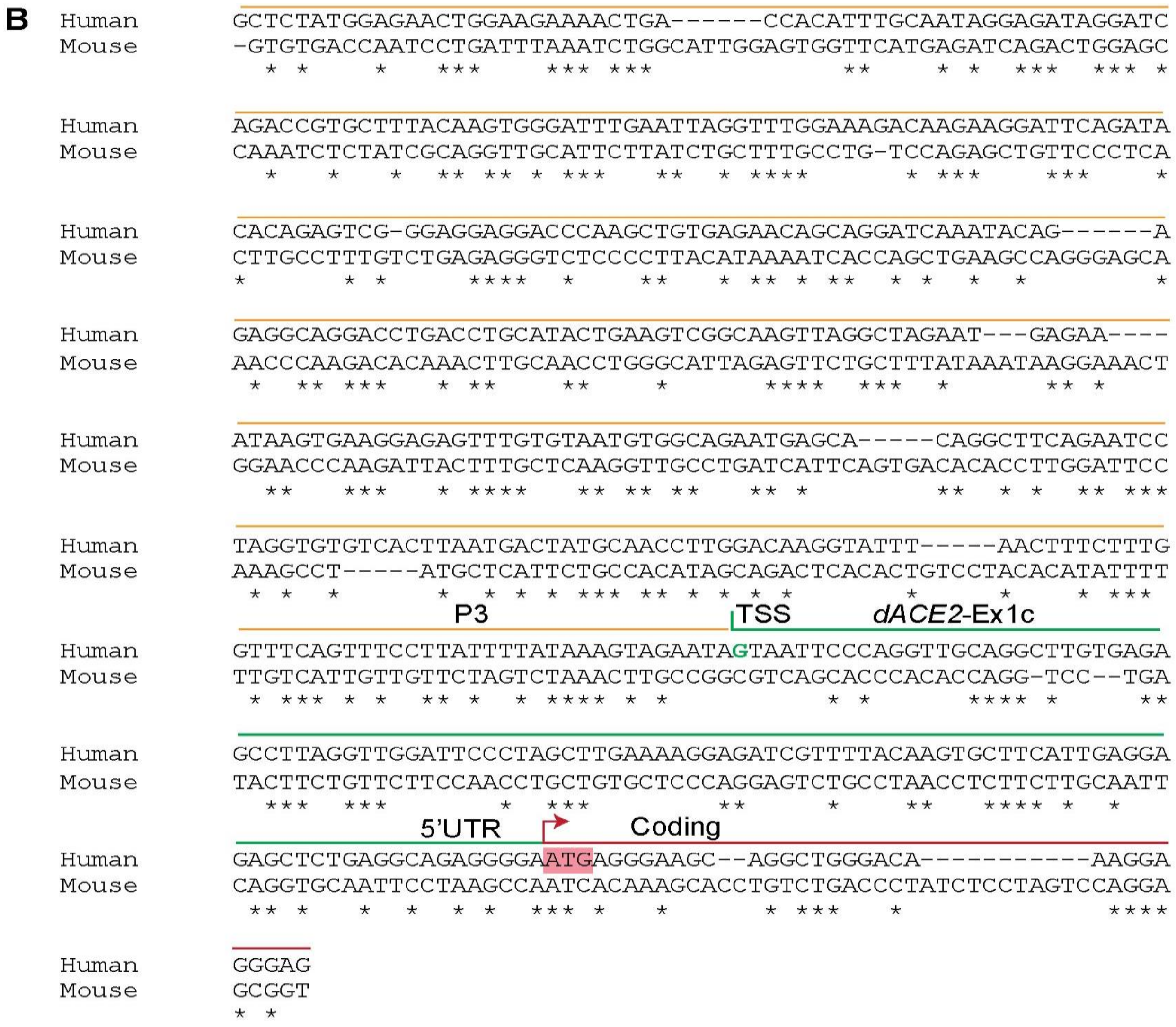

Figure S2. Conservation of the *dACE2*-Ex1c sequences.

A). Conservation of the 140 bp sequence of *dACE2*-Ex1c (human chrX:15,580,281-15,580,420, GRCh38/hg38) was analyzed by BLAT in 100 vertebrate species in the UCSC genome browser (www.genome.ucsc.edu). The sequence is highly conserved in primates but is less conserved or absent in non-primates, precluding *dACE2* transcript initiation or translation into an ACE2-type protein. The long bar indicates the entire Ex1c (140 bp) and the short bar indicates the protein-coding part of this exon (30 bp), starting from the ATG codon indicated by an arrow and highlight; the gene direction is from right to left. **B**). Comparison between human and mouse sequences; *- conserved bases; transcription start site (TSS) and translation start site (ATG) are indicated based on the human sequence. Human and mouse sequences share 43.7% identity within 500 bp (includes Ex1c, 5’UTR and promoter). Sequences were downloaded from UCSC genome browser and aligned using Clustal Omega.

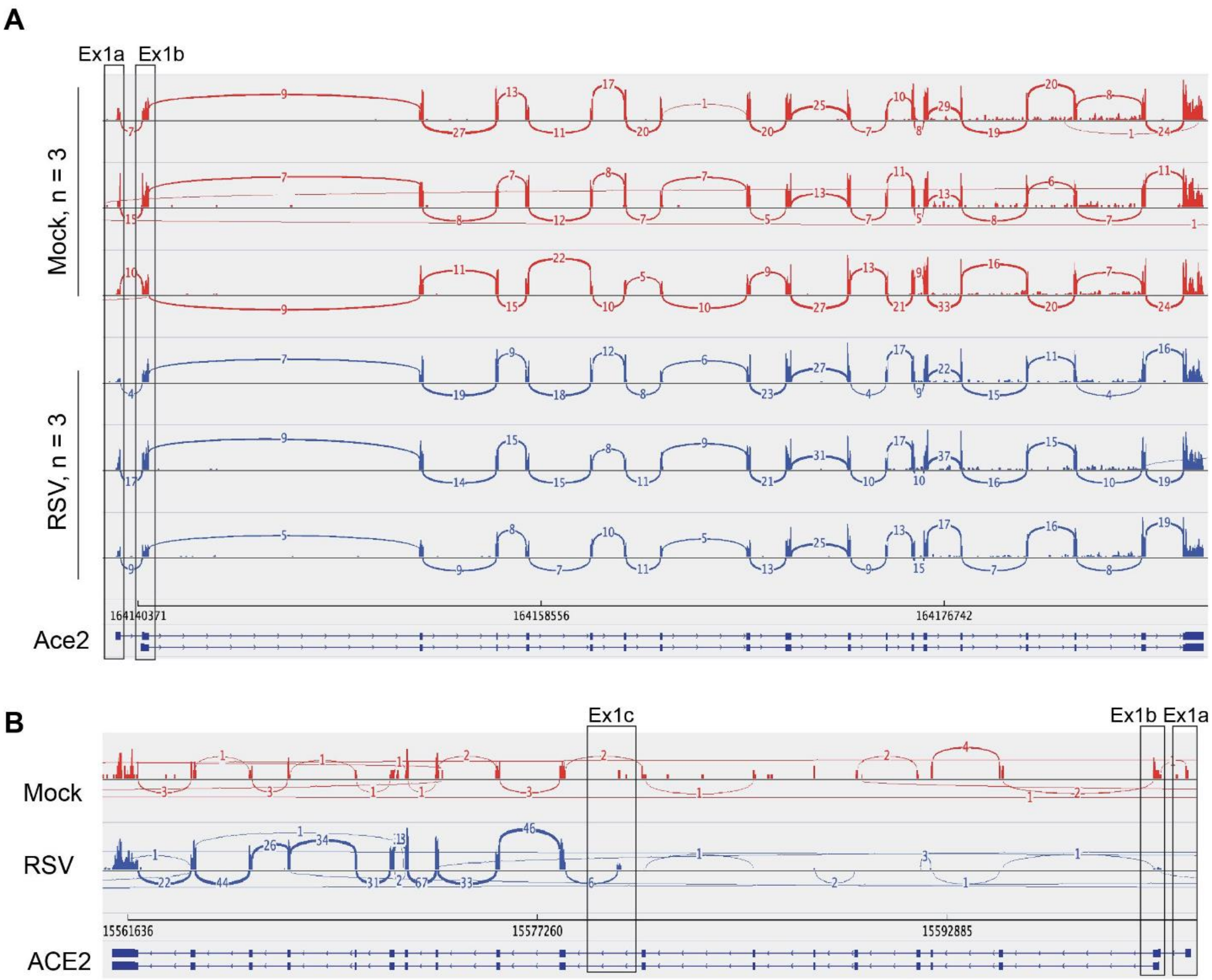

Figure S3. ***ACE2*** **expression patterns in mouse and human lung cells infected with the respiratory syncytial virus (RSV).**

A) Sashimi plots of the *Ace2* region in a lung RNA-seq dataset from mice mock/RSV- infected (in triplicates). *Ace2*-Ex1a and Ex1b show similar expression patterns in all samples. The expression of *dACE2*-Ex1c is not observed, consistent with the absence of the corresponding genomic sequence in mice (**Figure 1D**, **Figure S1A, B**). **B**) Sashimi plots of the *ACE2* region in H292, a human lung mucoepidermoid pulmonary carcinoma cell line, show that expression of *ACE2* from Ex1a and Ex1b and *dACE2* from Ex1c is very low at baseline. Only *dACE2* expression is induced by RSV infection. Note: The mouse and human *ACE2* genes are shown in opposite orientations, as presented in the Integrative Genomic Viewer (IGV). Dataset: PRJNA588982.

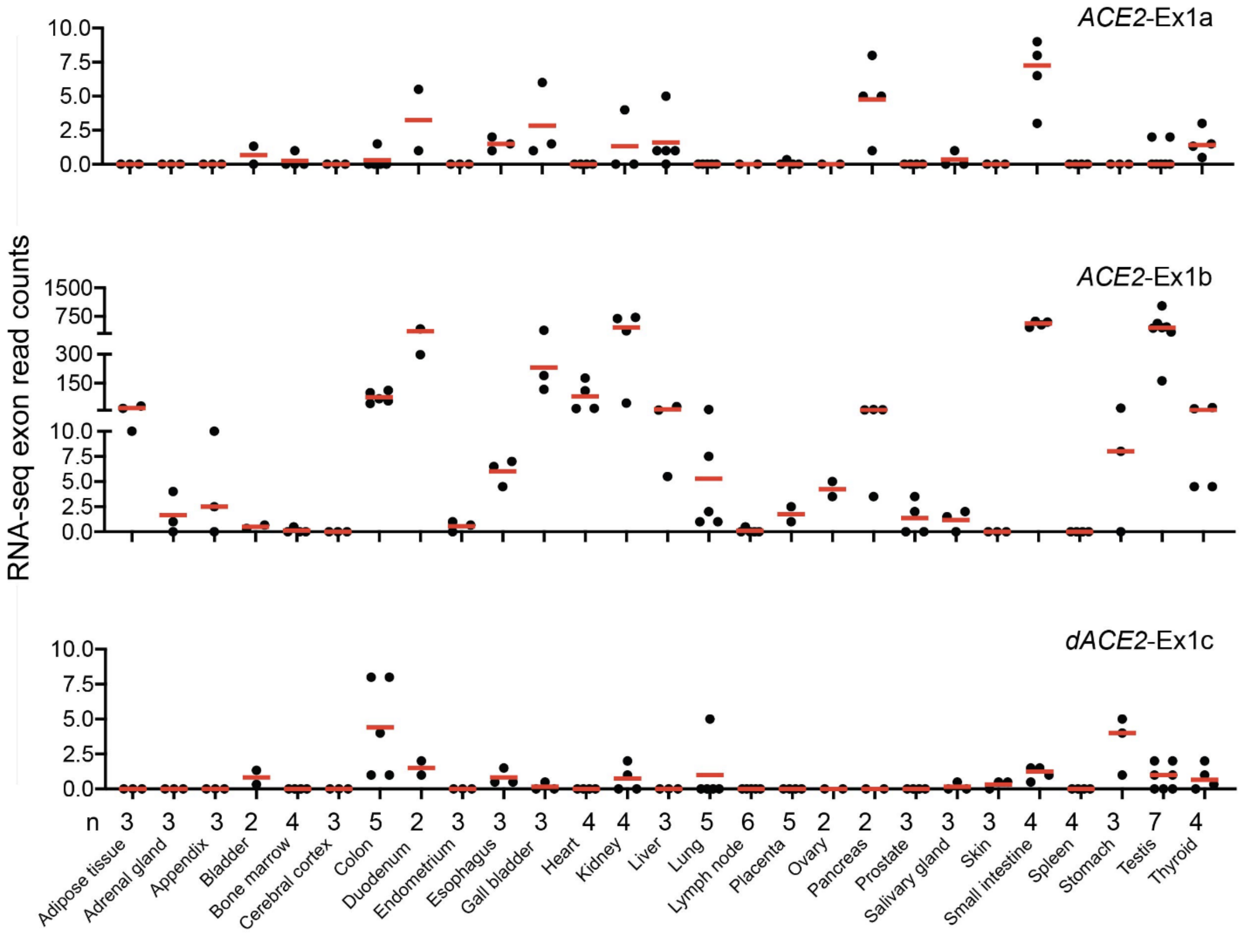

Figure S4. Expression of *ACE2* and *dACE2* in normal human tissues. RNA-seq read counts for *ACE2*-Ex1a and Ex1b and *dACE2*-Ex1c in 27 human tissues. Dataset: PRJEB4337, n = 95

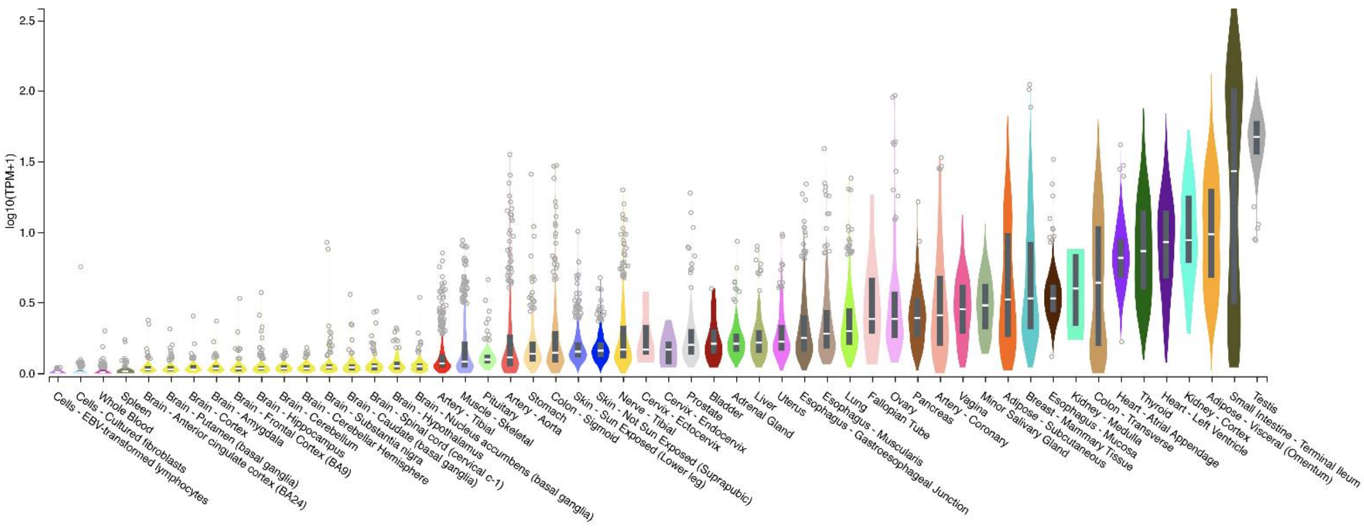

Figure S5. ***ACE2*** **expression in the Genotype-Tissue Expression (GTEx) project.**

Gene-based *ACE2* expression (combines *ACE2* and *dACE2* isoforms) in 17,382 normal human tissue samples of 54 tissue types in GTEx https://www.gtexportal.org/home/gene/ACE2.

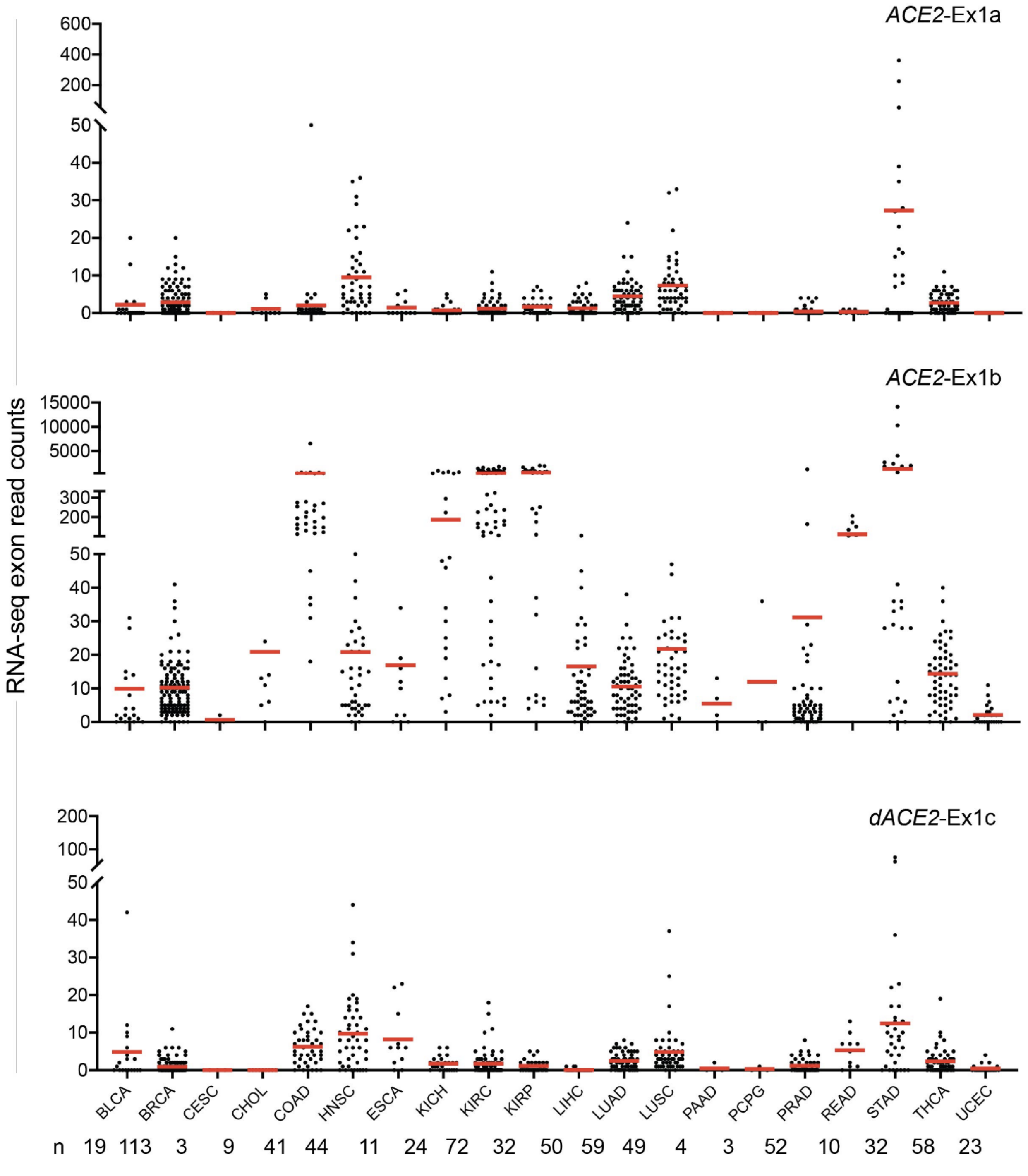

Figure S6. Expression of *ACE2* and *dACE2* in tumor-adjacent normal tissues in TCGA. Based on RNA-seq read counts, *ACE2*-Ex1b is detectable in multiple samples of several tissue types. *dACE2*-Ex1c expression is more restricted and most common in normal tissue adjacent to tumors of head and neck (HNSC), stomach (STAD), lung squamous carcinoma (LUSC), colon (COAD), and esophagus (ESCA).

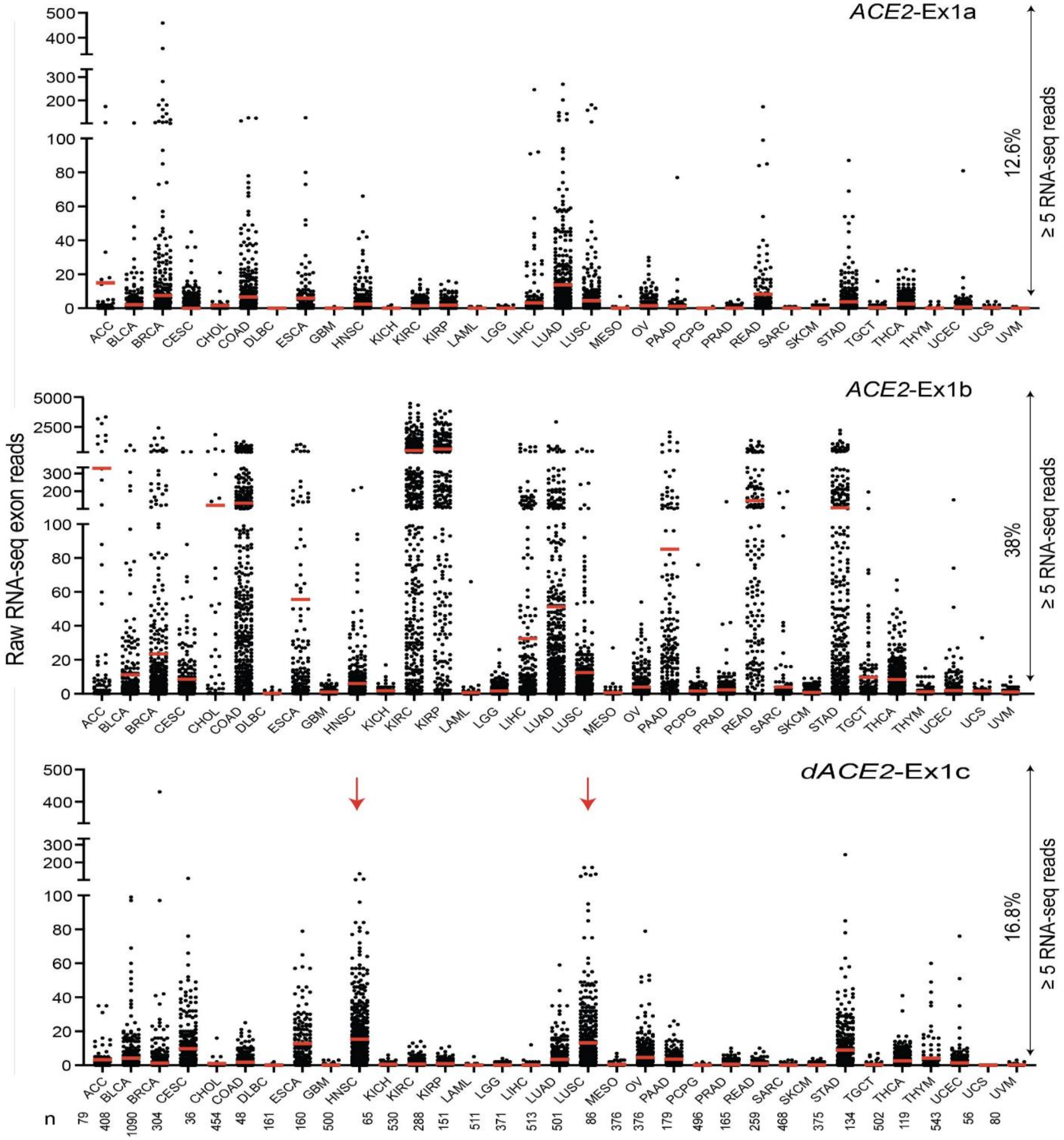

Figure S7. Expression of *ACE2* and *dACE2* across 10,185 tumors of 33 types in TCGA. Based on RNA-seq read counts, *ACE2*-Ex1b is most expressed in kidney tumors - kidney renal clear cell carcinoma (KIRC) and kidney renal papillary cell carcinoma (KIRP). Most samples expressing *dACE2*-Ex1c are squamous tumors of head and neck (HNSC) and the lungs (LUSC). Based on ≥5 reads/sample threshold, *ACE2*-Ex1a is expressed in 12.6%, *ACE2*-Ex1b – in 38.0% and *dACE2*-Ex1c - in 16.8% of all tumors.

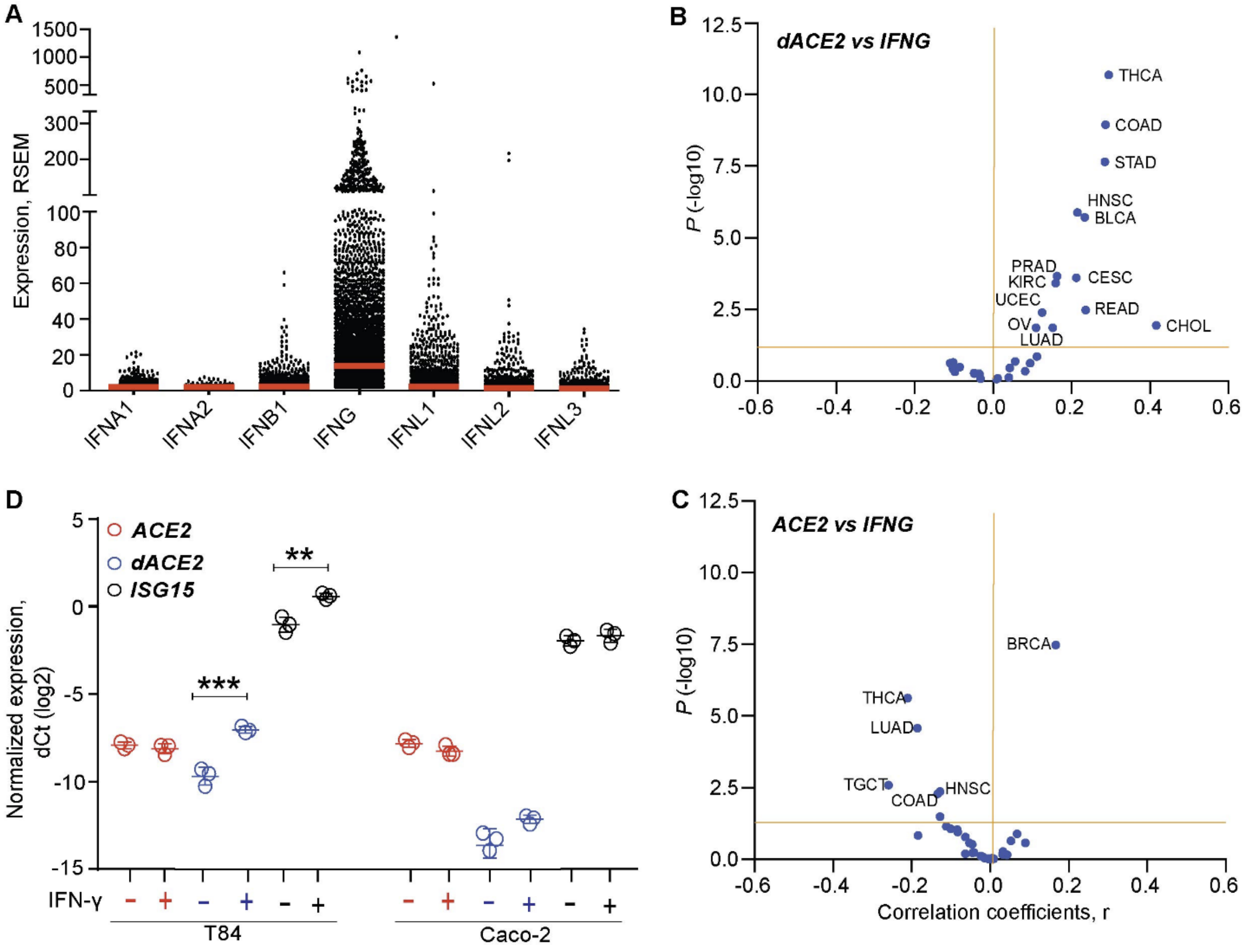

Figure S8. Analysis of *dACE2* and *ACE2* expression in relation to *IFNG* expression in TCGA tumors and *in vitro* IFN-ɣ treatment.

A) Expression levels of all *IFN* genes annotated in TCGA tumors (n = 10,185) were acquired from cBioPortal (https://www.cbioportal.org/); expression of *IFNL4* was not available. At RSEM ≥ 1, only expression of *IFNG* is common (61% samples), with mean expression RSEM=19.8 compared to other IFN genes (mean expression RSEM ≤ 1.3). **B**, **C)** Pearson correlation coefficients (r) for *dACE2* and *ACE2* vs. *IFNG* expression across tumors. *dACE2* showed significant positive correlations (r ≥ 0.2) with *IFNG* in 8 tumor types, while *ACE2* showed mainly negative correlations and only one positive correlation in breast cancer (r = 0.15). Expression values for *dACE2* and *ACE2* were based on log2 normalized exon read counts (Ex1b and Ex1c) and for *IFNG* - on RSEM values. **D)** Treatment of cell lines with IFN-ɣ (2ng/ml, 48 hrs) induced expression of *dACE2* but not *ACE2* in T84 cells.

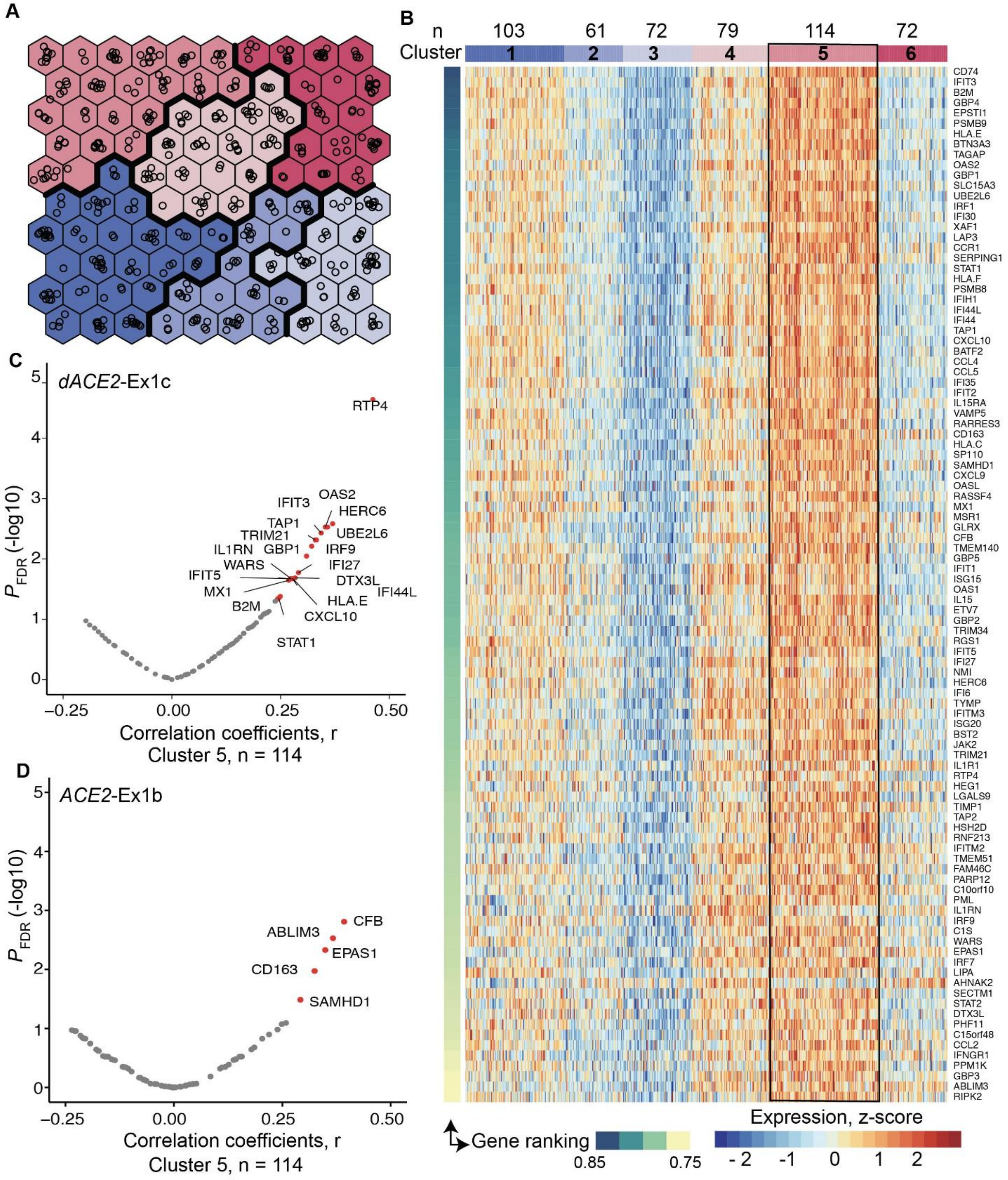

Figure S9. Unsupervised self-organizing map (SOM) analysis in TCGA-LUSC tumors

A) Construction of the unsupervised SOM of TCGA-LUSC tumors (n=501) based on Z-scores calculated for each of the 270 curated ISGs. Each hexagon includes a mean of 5 (range 1-14)

tumors with similar ISG expression profiles. Colors denote clusters (1-6) of tumors with similar ISG expression profiles. **B)** Heatmap of the 6 SOM-defined clusters plotting the expression of top 100 ISGs selected by ranking of the initial set of 270 ISGs based on their contribution to these clusters. Cluster 5 includes 114 tumors with the highest ISG expression, whereas cluster 3 includes 72 tumors with the lowest ISG expression. **C)** Volcano plots showing FDR-adjusted p-values and Pearson correlation coefficients (r) for expression of *dACE2* and *ACE2* in relation to expression of the top 100 ISGs within cluster 5. In total, *dACE2* was significantly (FDR p-value < 0.05) correlated with expression of 20 ISGs and *ACE2* - with 5 ISGs.

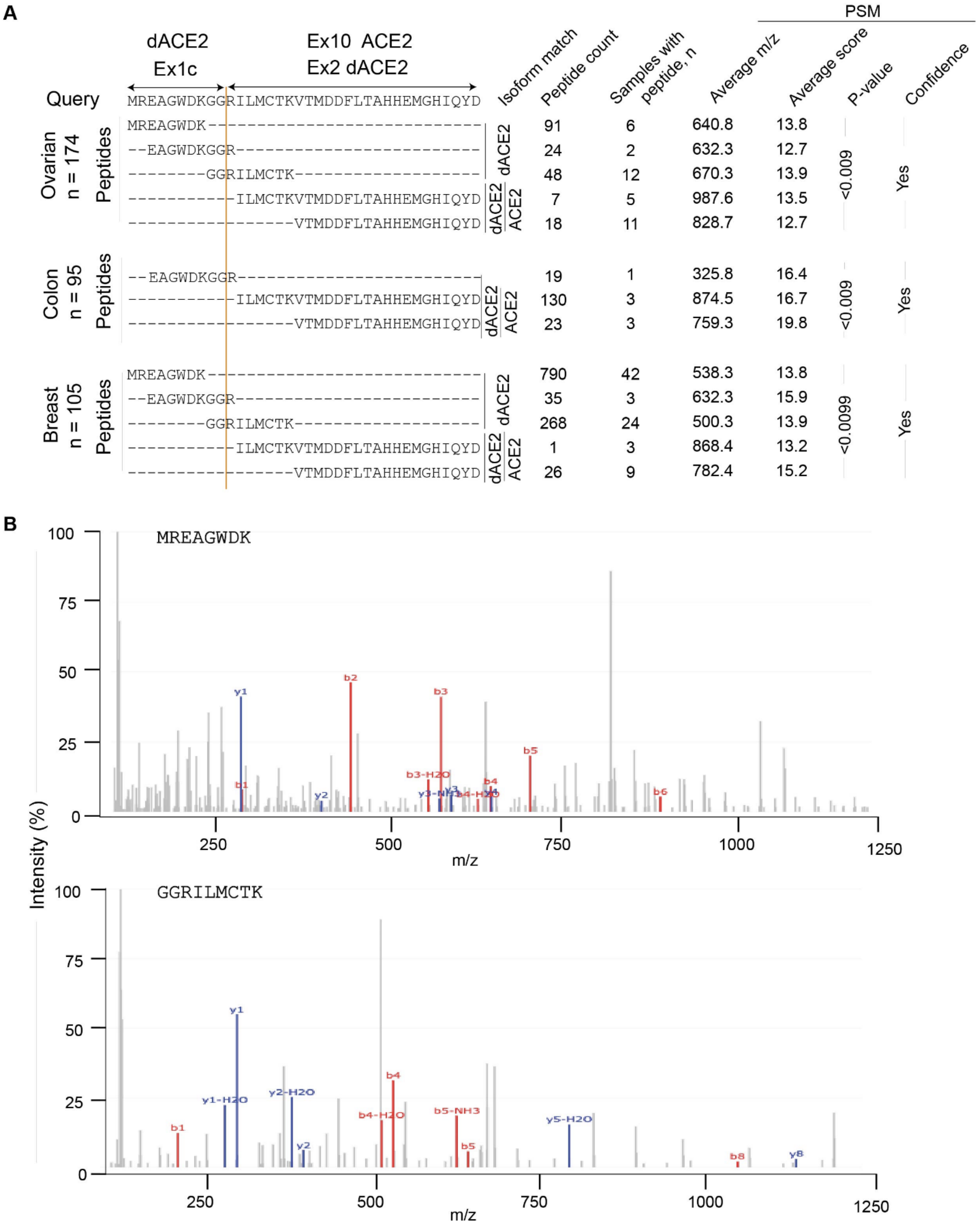

Figure S10. Peptides encoded by d*ACE2*-Ex1c are detected by protein sequencing in tumors.

A) Results of peptide query in PepQuery2 proteomics database of mass-spec data in 174 ovarian, 95 colon, and 105 breast tumors in TCGA (Wen et al., 2019). Three peptides – MREAGWDK, EAGWDKGGR, and GGRILMCTK uniquely correspond to 10 aa encoded by *dACE2*-Ex1c. The latter peptide results from the splicing of *dAC2*-Ex1c with its downstream exon. The total number of identified peptides, the number of samples with specific peptides, and corresponding parameters for a peptide-spectrum match (PSM) are shown in table format. **B)** Representative spectra of two peptides matching with the protein encoded by *dACE2*-Ex1c. M/z refers to the mass by charge ratio. The b-series and y-series ions showed the correct mapping of residues in the query aa sequence.

REFERENCES

Fagerberg, L., Hallstrom, B.M., Oksvold, P., Kampf, C., Djureinovic, D., Odeberg, J., Habuka, M., Tahmasebpoor, S., Danielsson, A., Edlund, K., *et al.* (2014). Analysis of the human tissue-specific expression by genome-wide integration of transcriptomics and antibody-based proteomics. Mol Cell Proteomics *13*, 397-406.

McAllister, C.S., Ansaldi, D., Growcott, E.J., Zhong, Y., Quackenbush, D., Wolff, K.C., Chen, Z., Tanaseichuk, O., Lelais, G., Barnes, S.W., *et al.* (2020). Dexamethasone inhibits respiratory syncytial virus-driven mucus production while increasing viral replication without altering antiviral interferon signaling. Virology *540*, 195-206.

Santer, D.M., Minty, G.E.S., Golec, D.P., Lu, J., May, J., Namdar, A., Shah, J., Elahi, S., Proud, D., Joyce, M., *et al.* (2020). Differential expression of interferon-lambda receptor 1 splice variants determines the magnitude of the antiviral response induced by interferon-lambda 3 in human immune cells. PLoS Pathog *16*, e1008515.

Stanifer, M.L., Kee, C., Cortese, M., Zumaran, C.M., Triana, S., Mukenhirn, M., Kraeusslich, H.G., Alexandrov, T., Bartenschlager, R., and Boulant, S. (2020). Critical Role of Type III Interferon in Controlling SARS-CoV-2 Infection in Human Intestinal Epithelial Cells. Cell Rep, 107863.

Wen, B., Wang, X., and Zhang, B. (2019). PepQuery enables fast, accurate, and convenient proteomic validation of novel genomic alterations. Genome Res *29*, 485-493.
